# Stain Normalization of Histopathological Images Based on Deep Learning: A Review

**DOI:** 10.3390/diagnostics15081032

**Published:** 2025-04-18

**Authors:** Chuanyun Xu, Yisha Sun, Yang Zhang, Tianqi Liu, Xiao Wang, Die Hu, Shuaiye Huang, Junjie Li, Fanghong Zhang, Gang Li

**Affiliations:** 1 School of Computer & Information Science, Chongqing Normal University, Chongqing 401331, China; xcy@cqnu.edu.cn (C.X.); 2022210516084@stu.cqnu.edu.cn (Y.S.);; 2 School of Artificial Intelligence, Chongqing University of Technology, Chongqing 401135, China; 3 National Center for Applied Mathematics, Chongqing Normal University, Chongqing 401331, China

**Keywords:** histopathological image analysis, stain normalization, computational pathology, deep learning

## Abstract

Histopathological images stained with hematoxylin and eosin (H&E) are crucial for cancer diagnosis and prognosis. However, color variations caused by differences in tissue preparation and scanning devices can lead to data distribution discrepancies, adversely affecting the performance of downstream algorithms in tasks like classification, segmentation, and detection. To address these issues, stain normalization methods have been developed to standardize color distributions across images from various sources. Recent advancements in deep learning-based stain normalization methods have shown significant promise due to their minimal preprocessing requirements, independence from reference templates, and robustness. This review examines 115 publications to explore the latest developments in this field. We first outline the evaluation metrics and publicly available datasets used for assessing stain normalization methods. Next, we systematically review deep learning-based approaches, including supervised, unsupervised, and self-supervised methods, categorizing them by core technologies and analyzing their contributions and limitations. Finally, we discuss current challenges and future directions, aiming to provide researchers with a comprehensive understanding of the field, promote further development, and accelerate the progress of intelligent cancer diagnosis.

## 1. Introduction

Cancer is the second leading cause of death globally and a significant barrier to increasing life expectancy, with incidence and mortality rates rising each year. According to the World Health Organization (WHO), the number of global cancer cases may increase by 60% over the next 20 years, presenting a severe challenge for prevention and control [1]. Therefore, early detection and treatment of cancer are crucial for global cancer management. Currently, histopathological diagnosis is still regarded as the “gold standard” for diagnosing almost all cancers and a wide range of diseases, as it provides a direct visualization of cancer aggressiveness at the cellular level [2]. This diagnosis relies on hematoxylin and eosin (H&E)-stained tissue sections, where hematoxylin highlights nuclei (blue-purple) and eosin marks the cytoplasm (pink), enabling clear visualization of tissue morphology [3].

**Figure 1:**
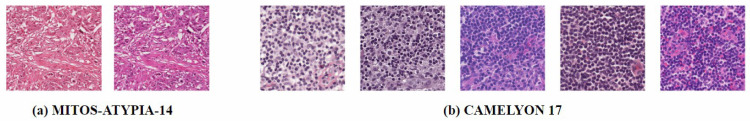
Stain variation. (**a**) MITOS-ATYPIA-14 dataset [10]: The same glass slide scanned by two different scanners, resulting in digital slides with different color distributions. (**b**) CAMELYON 17 dataset [11]: This dataset contains slides from five different centers, and the five distinct styles have caused color distribution shifts.

In the past, pathologists typically examined histopathological images through microscopes, which was a highly labor-intensive process. With the advent of whole-slide scanners and computer-aided diagnosis (CAD) systems, more pathology laboratories are gradually transitioning to digital workflows [4]. However, the digital histopathological image production process is complex and fraught with issues. In multicenter studies, data from different hospitals often exhibit staining differences, as shown in Figure 1 [5]. Laboratories rarely document exact staining parameters like concentrations, temperatures, and dye batches. Even when protocols are identical, scanner instrument errors such as white balance and lighting problems can cause variations [6]. This not only leads to inconsistent data distributions but also significantly impacts the performance and robustness of CAD systems [7]. In real-world scenarios, especially in retrospective studies, it is nearly impossible to restore the original parameters for each slide. Without normalization, the presence of artificial noise from color variations reduces the accuracy of AI algorithms in medical image analysis [8]. Additionally, scanning artifacts such as “yellowness” from aging slides or uneven lighting affect image quality, making it difficult for pathologists to clinically interpret and compare slides with different contrasts. Although retraining algorithms to handle color shifts is possible, building training datasets for different environments is extremely costly [9].

**Figure 2:**
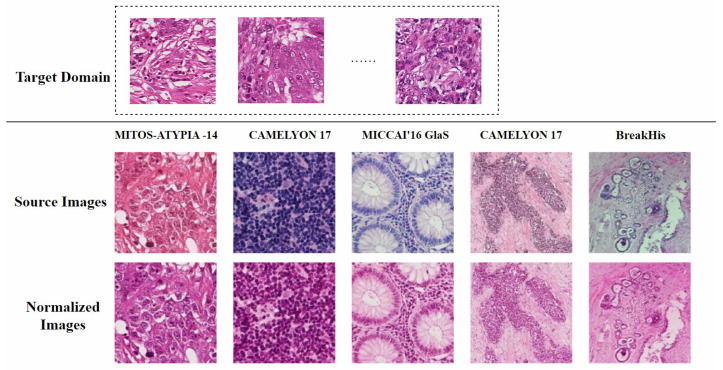
Conceptual diagram of stain normalization. The first row represents the target domain, a set of images with relatively uniform staining colors. The second row shows the source domain, with varying staining styles. The third row displays the normalized source domain images, which have adopted the staining style of the target domain while retaining the tissue structure of the source domain. The figure is adapted from [14].

A feasible solution is to standardize all H&E-stained images to the color distribution of the training set to minimize the impact of color variations on subsequent computational processes [12]. This process is known as stain normalization. Therefore, stain normalization addresses a practical problem: how to automatically adapt images from different sources to a single representation while preserving diagnostically relevant features. As shown in Figure 2, the target domain for stain normalization can be defined as a set of images with relatively uniform staining colors, while the source domain includes all other images. The process of stain normalization requires adjusting the color distribution of source domain images to match that of the target domain images [13]. Meanwhile, it is essential to preserve the original tissue structure as much as possible and avoid introducing artifacts.

In addition, while some modern studies explore alternatives to RGB image recognition technology, like switching to multispectral microscopy or IR imaging to overcome its drawbacks, these methods are incompatible with existing histopathology infrastructure and established clinical standards [15]. Shifting to new methods requires revising diagnostic criteria, long-term validation of new markers, and abandoning access to millions of archived RGB slides (the basis for diagnosis in the past 50 years), and poses legal risks, potentially leading to misdiagnosis and litigation. Therefore, stain normalization remains irreplaceable in current RGB-based medical diagnosis.

Existing research on stain normalization is typically divided into two major categories: traditional methods and deep learning methods. Traditional methods include color-matching-based approaches and stain-separation-based approaches. In color-matching methods, the source image is typically aligned with the target image by matching the mean and standard deviation in the color space, while stain separation methods attempt to independently separate and standardize each staining channel in the optical density (OD) space [16]. Most traditional stain normalization methods rely on reference images to compute stain statistics, and if the selected reference image does not represent the color distribution of the target domain, the normalization result can significantly deviate [17]. In recent years, with the advancement of deep learning technology, the research direction for stain normalization methods has undergone a significant shift. Increasingly, approaches are adopting deep learning techniques such as Generative Adversarial Networks (GANs) [18], Autoencoders (AEs) [19], and Diffusion Models (DMs) [20]. These deep learning-based methods have gained popularity due to their ability to eliminate the need for complex preprocessing, their independence from reference templates for normalization, and their strong robustness and adaptability, making their application prospects and research interest continue to rise.

Although deep learning methods show great promise in the field of stain normalization, there are currently few review studies specifically focused on these methods. To fill this gap, this study provides a review of the relevant literature. The structure of this study is organized as follows: Section 2 introduces the tissue preparation and digitization process, which helps researchers better understand the sources of stain variation. Section 3 describes the materials and methods employed in this study. Section 4 elaborates on the commonly used evaluation metrics and representative datasets in stain normalization research. In Section 5, these deep learning methods are categorized as supervised, unsupervised, and self-supervised approaches based on the data labeling and availability, and further classified according to core technologies, highlighting their main contributions and limitations. Section 6 discusses the current issues and challenges in this field, pointing out potential improvements for future research to serve as a reference for researchers. Finally, Section 7 provides a conclusion of this study.

## 2. Tissue Preparation and Digitization Process

During the tissue preparation process, important factors that affect H&E staining include dye concentration, mordant ratio, pH levels, oxidation, and staining time [21]. Despite the variety of staining protocols, no consensus has been reached among pathologists—some prefer a darker stained background, while others favor a clean background with sharp nuclear staining [21]. While these color variations may have minimal impact on pathologists, they can reduce the performance of CAD systems. Understanding the preparation process helps us better comprehend the sources of staining variation, enabling more evidence-based scientific research. Below are some basic preparation steps:

### 2.1. Specimen Collection and Fixation

Once the specimen is collected by the clinician, it should be promptly immersed in a fixative solution (usually 10% formalin) to permeate the tissue and provide a certain level of hardness [22]. Fixation time depends on the specimen’s size. If fixation lasts too long, it may result in over-fixation and even affect staining [23].

### 2.2. Dehydration and Clearing

Dehydration is achieved through a graded series of alcohol solutions, where the tissue block passes through 70%, 80%, 95% I, 95% II, 100% I, and 100% II alcohol solutions. The purpose of dehydration is to remove excess water from the tissue specimen [23]. Xylene is then used as a clearing agent to eliminate any remaining alcohol, as paraffin will later be infiltrated into the tissue, but water and alcohol do not mix with paraffin, thus necessitating the use of a clearing agent [24].

### 2.3. Paraffin Infiltration and Embedding

The tissue is infiltrated with hot paraffin at 56–58 °C, which hardens to provide support for the tissue [25]. The tissue is then placed in an embedding mold and liquid paraffin is poured in, which cools to form a paraffin block [22].

### 2.4. Sectioning

The paraffin-embedded tissue block is sectioned using a microtome. The section thickness depends on the cellular thickness, with conventional staining sections typically not exceeding 6 µm. The smooth, shiny side of the section is then affixed to a glass slide [24].

### 2.5. Staining

After sectioning, the tissue sample appears translucent, so stains are applied to enhance contrast and highlight important tissue features [24]. The most commonly used staining method is H&E staining: hematoxylin stains the basophilic structures (mainly the cell nucleus and genetic material) blue-purple, while eosin stains the acidophilic structures (primarily the cytoplasm and cell membrane) pink, making the cell and tissue morphology clearly visible [24]. Variations in staining time or tissue temperature during staining may lead to staining differences.

### 2.6. Mounting

Finally, a cover slip is applied over the tissue using neutral resin to preserve the slide permanently and facilitate microscopic examination [23].

### 2.7. Digitization

The prepared slides are scanned using a whole-slide scanner to generate Whole-Slide Images (WSIs), which can be viewed by pathologists and used for histopathological image analysis [26]. Even when the same set of slides is scanned, different scanners may produce variations in color. As shown in Figure 1, in the MITOS-ATYPIA-14 dataset [10], when scanning the same set of slides using the Aperio Scanscope XT (Leica Biosystems, Nussloch, Germany) and the Hamamatsu Nanozoomer 2.0-HT (Hamamatsu Photonics, Hamamatsu, Japan), the former produced images with an overall pink hue, while the latter had a more purple tone.

## 3. Materials and Methods

This study systematically reviews and analyzes research on deep learning-based stain normalization methods published between 2017 and 2024. We used popular databases such as Scopus (https://www.scopus.com, accessed on 25 March 2025) and Google Scholar (https://scholar.google.com, accessed on 25 March 2025), searching for relevant papers using keywords such as “deep learning” combined with “stain normalization”, “color normalization”, and “stain standardization”. Some papers were also found through citation or reference tracking. Another principle was to select papers related to H&E staining as much as possible and to collect comprehensive relevant literature to help researchers conduct more informed scientific research.

## 4. Datasets and Evaluation Metrics

### 4.1. Datasets

#### 4.1.1. Datasets for Quantitative Evaluation of Stain Normalization

Quantitative evaluation of stain normalization results typically requires paired datasets, but in practice, such data are difficult to obtain. Most methods use the MITOS-ATYPIA-14 dataset (16 WSIs in total) for quantitative evaluation. This dataset is part of the MITOS-ATYPIA-14 challenge (https://mitos-atypia-14.grand-challenge.org/, accessed on 25 March 2025) and contains histological images of breast cancer tissue, which can be used for the evaluation of mitosis detection and nuclear atypia scoring. The dataset includes 11 slides for training and 5 slides for testing. These slides are stained with standard H&E and scanned using two scanners: Aperio Scanscope XT (Leica Biosystems, Nussloch, Germany) and Hamamatsu Nanozoomer 2.0-HT (Hamamatsu Photonics, Hamamatsu, Japan). For each slide, pathologists selected several frames at 10×, 20×, and 40× magnifications within the tumor region. Rigid registration is required before use to eliminate any misalignment.

#### 4.1.2. Classification and Segmentation Datasets

Due to the scarcity of paired datasets for direct quantitative evaluation of stain normalization, researchers typically validate the effectiveness of stain normalization by assessing its improvement on the performance of downstream tasks, such as classification and segmentation. Currently, commonly used public classification datasets in this field include CAMELYON 16 (400 WSIs total) [27] and CAMELYON 17 (1000 WSIs total) [11], while frequently used public segmentation datasets include MICCAI’2015 GlaS (165 patches total) [28] and MoNeSeg (30 patches total) [29]. Detailed information on representative public datasets is shown in Table 1.

### 4.2. Evaluation Metrics

After performing stain normalization on histopathological images, it is essential to effectively evaluate the quality of the images. This includes assessing the staining similarity between the normalized images and the target images, as well as evaluating the preservation of tissue structure between the normalized images and the source images. Such evaluations help to highlight the strengths and weaknesses of various stain normalization algorithms, thereby providing guidance for the development of new methods. Commonly used evaluation metrics include the following:

#### 4.2.1. Peak Signal-to-Noise Ratio (PSNR)

PSNR is a commonly used metric for objectively evaluating image quality and is widely applied in the field of image processing. It measures image quality by calculating the pixel-level errors between images, and its formula is as follows:

(1) $$PSNR=10\log_{10}\left(\frac{{\left(2^{n}-1\right)}^{2}}{MSE}\right)$$

where *n* represents the number of bits per pixel, and MSE is the mean squared error between the target image and the normalized image. The unit of PSNR is decibels (dB), and the higher the value, the lower the distortion of the normalized image and the better the quality. Generally, a PSNR value greater than 30 dB indicates that the normalized image has good quality. However, the PSNR metric has certain limitations. It only considers the error between pixels at the same locations in two images and does not take into account the visual characteristics of the human eye, which may result in discrepancies between its evaluation and human visual perception.

#### 4.2.2. Structural Similarity Index (SSIM)

SSIM [34] is a perceptual image similarity metric that primarily focuses on changes in structural information, rather than simply calculating absolute errors like PSNR. SSIM evaluates the similarity of two images by comparing their luminance, contrast, and structural information within small local windows. Let *x* and *y* represent the two images to be tested, i.e., one normalized image and one target image. The SSIM value for each local window is calculated as follows:

(2) $$SSIM\left(x,y\right)=\frac{\left(2\mu_{x}\mu_{y}+C_{1}\right)\left(2\sigma_{xy}+C_{2}\right)}{\left(\mu_{x}^{2}+\mu_{y}^{2}+C_{1}\right)\left(\sigma_{x}^{2}+\sigma_{y}^{2}+C_{2}\right)}$$

where $\mu_{x}$ is the mean of *x*, $\mu_{y}$ is the mean of *y*, $\sigma_{x}^{2}$ is the variance of *x*, $\sigma_{y}^{2}$ is the variance of *y*, and $\sigma_{xy}$ is the covariance between *x* and *y*. $C_{1}={\left(k_{1}L\right)}^{2}$ and $C_{2}={\left(k_{2}L\right)}^{2}$ are constants used to maintain stability. *L* is the dynamic range of pixel values, with $k_{1}=0.01$ and $k_{2}=0.03$. The SSIM value ranges from 0 to 1, and a higher SSIM indicates better stain normalization performance.

#### 4.2.3. Normalized Median Intensity (NMI)

NMI [35] quantifies the brightness distribution of an image by calculating the ratio of the median intensity to the 95th percentile intensity. The formula is as follows:

(3) $$NMI\left(I_{in}\right)=\frac{Median\{u(t)\}}{P_{95}\left\{u\left(t\right)\right\}}$$

where u(t) represents the average value of the RGB channels of the image, Median denotes the median, and $P_{95}$ signifies the 95th percentile value.

NMI is capable of effectively assessing the color consistency of images following stain normalization, particularly when normalizing across different staining styles. It aids in determining whether the normalized images have preserved the brightness distribution of the target domain. NMI exhibits insensitivity to noise and local variations within the image, rendering it suitable for evaluating the global color distribution post-normalization. However, NMI primarily focuses on brightness distribution and is unable to directly assess structural information or color details in the image.

#### 4.2.4. Pearson Correlation Coefficient (PCC)

PCC [36] is used to measure the linear correlation between two images. Its value ranges from [−1, 1], where 1 indicates a perfect positive correlation, −1 indicates a perfect negative correlation, and 0 indicates no correlation. The formula is as follows:

(4) $$PCC=\frac{\sum_{i}\left(x_{i}-\mu_{x}\right)\left(y_{i}-\mu_{y}\right)}{\sqrt{\sum_{i}{\left(x_{i}-\mu_{x}\right)}^{2}}\sqrt{\sum_{i}{\left(y_{i}-\mu_{y}\right)}^{2}}}$$

where $x_{i}$ and $y_{i}$ represent the pixel values of the two images, and $\mu_{x}$ and $\mu_{y}$ denote the mean values of the two images, respectively.

PCC is effective in evaluating the linear relationship between the normalized image and the target image, making it suitable for assessing whether the color distribution of the normalized image aligns with that of the target image. PCC is straightforward to compute, and its results are intuitive, making it ideal for quickly evaluating the effectiveness of stain normalization. However, PCC can only measure linear relationships and is unable to capture nonlinear relationships or structural information. Moreover, its accuracy and effectiveness rely on the assumption that the data follow (or approximately follow) a normal distribution. If the data significantly deviate from normality, the PCC results may become unreliable.

#### 4.2.5. Feature Similarity Index (FSIM)

FSIM [37] is a similarity metric based on image features, combining phase congruency and gradient magnitude information of the image. Its value ranges from [0, 1], where 1 indicates perfect similarity. The formula is as follows:

(5) $$FSIM=\frac{\sum_{x\in \Omega }S_{L}\left(x\right)\cdot S_{PC}\left(x\right)}{\sum_{x\in \Omega }S_{PC}\left(x\right)}$$

where $S_{L}\left(x\right)$ denotes local similarity, and $S_{PC}\left(x\right)$ represents phase congruency.

FSIM is effective in evaluating whether the structural information and detailed features of images are preserved after stain normalization, making it suitable for assessing whether the tissue structure of the normalized image aligns with that of the target image. FSIM combines phase congruency and gradient information, enabling it to capture multi-scale features of the image, and is thus well suited for evaluating the quality of details in stain-normalized images. However, FSIM has a relatively high computational complexity, particularly for high-resolution images, which results in longer computation times.

In summary, when choosing evaluation metrics, the specific research objectives and key points should be the primary basis. If the research focus is on accurately quantifying the consistency of color distribution, metrics like NMI and PCC, which focus on color distribution evaluation, are more suitable. If more attention is paid to the preservation of tissue structure, FSIM has more advantages. Secondly, consider the characteristics of the data. Factors such as data resolution and noise level also affect the selection of evaluation metrics. For high-resolution images, although FSIM has a high computational complexity, it can better evaluate their detailed features. For data with high noise, some noise-sensitive metrics may not be applicable.

#### 4.2.6. Analysis of the Clinical Relevance of Metrics

In the field of histopathological image stain normalization, metrics such as PSNR and SSIM are widely used to evaluate the effect of stain normalization. However, their correlations with clinical diagnosis are rather complex. Thoroughly exploring the clinical relevance of these metrics is of great significance for improving the accuracy of histopathological image analysis and its clinical application value.

PSNR mainly measures the image quality by calculating the pixel-level error. Theoretically, the higher its value, the less distorted the image is. Nevertheless, in practical clinical applications, a high PSNR value does not always ensure a good diagnostic result. For example, in the analysis of H&E-stained images of breast cancer tissues, a stain normalization method may significantly increase the PSNR value of the image, indicating a reduction in pixel-level errors [5]. However, it may cause the originally clear blue-purple color of the cell nuclei to be over-adjusted, resulting in unnatural color deviations. Such color changes can interfere with pathologists’ judgment of cell morphology and structure, thus affecting the accuracy of diagnosis.

In clinical practice, the observation of some fine tissue structures is crucial for disease diagnosis. If a method overemphasizes the consistency of the overall structure, some fine pathological structures may be blurred or lost during the processing. For instance, the small lesions of early-stage colon cancer may become less obvious due to this kind of processing, making it difficult for pathologists to accurately determine the pathological conditions. It may also cause CAD systems to miss such details [38]. This shows that although SSIM has certain advantages in measuring the structural similarity of images, overly pursuing this technical indicator may sacrifice some detailed information that is crucial for clinical diagnosis, thereby reducing the clinical utility.

NMI, PCC, and FSIM also have their own drawbacks. If the image is processed for stain normalization solely based on NMI, a situation may occur where the color consistency meets the standard, but the key structural features are weakened or lost. This is because NMI only focuses on the brightness distribution and ignores the complex structural information within the tissues. PCC can only reflect the linear correlation between images and is unable to capture the nonlinear pathological information. In clinical diagnosis, these nonlinear pathological features are essential for determining the type and severity of diseases. Therefore, the correlation between PCC and the clinical diagnosis results is not stable, and it cannot be solely relied on to evaluate the value of stain-normalized images for clinical diagnosis. Although FSIM can measure the overall structural similarity of images, during the calculation process, it may ignore some local fine pathological changes due to excessive emphasis on the overall features. Therefore, regarding the correlation between FSIM and the clinical diagnosis results, its computational efficiency and the ability to capture fine pathological changes need to be comprehensively considered, and it cannot be simply used to evaluate the practicality of stain-normalized images for clinical diagnosis.

Therefore, when conducting research, researchers should not merely focus on the high or low values of evaluation metrics. Instead, they need to closely consider the preservation effect of pathological features in the stain-normalized images. Special attention should be paid to avoiding the loss of critical diagnostic details due to the stain normalization process. After all, in the field of histopathological image stain normalization, each evaluation method has its inherent limitations. Thus, it is particularly crucial to comprehensively adopt multiple evaluation metrics and conduct in-depth analysis of their correlations with the actual staining effects. This not only helps to more comprehensively and accurately evaluate the advantages and disadvantages of stain normalization methods but also provides more valuable references for subsequent research and clinical applications.

## 5. Deep Learning-Based Stain Normalization in Histopathology

In recent years, the application of deep learning in medical image analysis has become increasingly widespread. This trend is driven by factors such as the use of high-performance Graphics Processing Units (GPUs) like the NVIDIA A100 (with 80 GB memory and 624 TFLOPS compute performance) or the RTX 4090 (24 GB memory, 82.6 TFLOPS), which are optimized for parallel processing of large-scale medical image datasets. The accumulation of extensive medical image data, coupled with the powerful representational capacity of deep learning models, has further accelerated progress. For many years, research on stain normalization in histopathology has continued in the field of medical imaging, and with the advancement of deep learning technologies, utilizing deep learning methods to address stain normalization has gradually become the mainstream trend. This section provides a detailed overview of stain normalization methods in histopathological image analysis based on deep learning. These methods are categorized as supervised, unsupervised and self-supervised, depending on the availability and labeling of data. Furthermore, based on the core technologies used, these methods are further divided into those based on GAN and other types of approaches.

### 5.1. Supervised Methods

Supervised learning refers to training a model using labeled data samples, where the model learns based on inputs and corresponding output labels [39]. In the field of stain normalization research, researchers utilize labeled data such as classification labels, segmentation masks, or paired data to precisely control and improve the staining process, thereby enhancing stain quality and consistency. This approach relies on accurate data labeling to ensure that the learning algorithm can effectively learn how to perform correct normalization from the input data. Table 2 summarizes supervised stain normalization methods.

#### 5.1.1. Generative Adversarial Network (GAN) Methods

**Figure 3:**
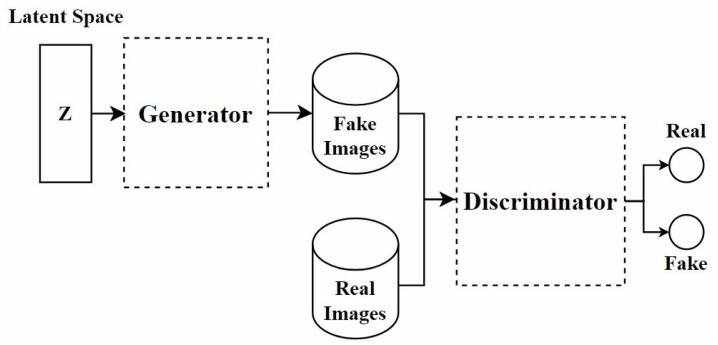
The architecture of the GAN model.

In recent years, GANs have been widely applied in the field of stain normalization. As shown in Figure 3, a GAN consists of two core components: the generator and the discriminator. The generator is responsible for generating images similar to real target images, while the discriminator determines whether these images are real or generated. This adversarial training is the core training method of GAN. They compete with each other. The generator strives to make the generated images more realistic, and the discriminator constantly improves its discrimination ability. Through this confrontation, the performance of both is continuously enhanced, and ultimately, the generator can produce images close to the real target. In the context of stain normalization for histopathological images, the standard-stained images are regarded as the target images. The images to be normalized are processed by the generator to generate images with the standard staining style. The discriminator judges the generated images. If the generated images can deceive the discriminator, the goal of stain normalization is achieved. In this process, both the generator and the discriminator are constantly improved, making the generated images more and more in line with the standard. The ultimate goal is to generate images that retain the structure of the source image while exhibiting the staining characteristics of the target image.

**Figure 4:**
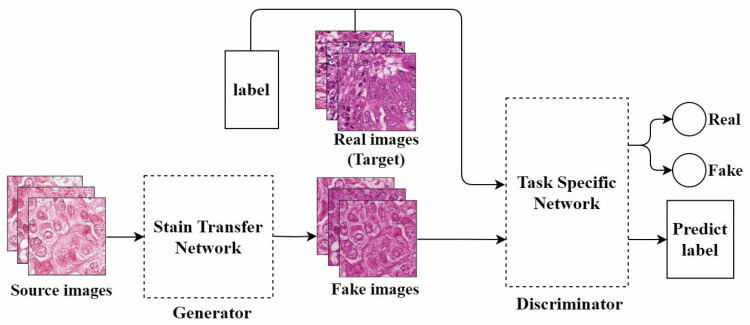
The model architecture proposed by Bentaieb et al., adapted from [40].

Bentaieb et al. [40], from the perspective of style transfer [48], proposed the first GAN model specifically designed for stain normalization. As shown in Figure 4, this model was the first to apply fully trainable normalization and image analysis techniques within the same framework. It uses a supervised learning approach, trained on data with classification labels or segmentation masks. The network consists of two parts: One part focuses on stain transfer, which converts the source image into one that is similar to the target image in terms of staining appearance while preserving the structural content of the source image. The other part is a task-specific network responsible for determining the authenticity of the image and predicting classification or segmentation labels, used for adversarial training. Although this method is effective, it has certain limitations in generating images with specific staining characteristics.

The High-Resolution Network (HRNet) [49] is an advanced deep learning architecture that excels in tasks requiring the preservation of high-resolution features. Inspired by this, Nishar et al. [41] proposed a stain transfer generator network based on HRNet. This network requires less training time and can enhance its generalization ability using only a small number of paired images with reference stains and test stains.

Liang et al. [42] proposed two stain style transfer models based on GAN: SSIM-GAN and DSCSI-GAN. To train these networks, they used a pre-trained classifier network to calculate feature preservation loss and innovatively employed the structural similarity index matrix and the directional statistics-based color similarity index (DSCSI) [50] as loss functions in the image reconstruction process. However, the color information in this method may become a form of noise, causing the model to overly focus on the color details of the training data. This could reduce the model’s generalization ability when handling images from different data sources.

Kausar et al. [43] proposed a model called stain acclimation generative adversarial network (SA-GAN), which consists of one generator and two discriminators. In this model, a reference template image selected by pathologists is used, and color attribute metrics are extracted from it to train the network. This is the first stain transfer method to combine color attribute details with a deep GAN. However, treating the color pattern of an image as a style for conversion may introduce unnecessary artifacts [51].

**cGAN:** The conditional Generative Adversarial Network (cGAN) [52] is a variant of GAN that introduces conditional information. Simply put, cGAN not only takes in a noise vector but also additional conditional information (such as labels or specific features) to control certain attributes of the generated data. This allows the generator to utilize these conditions to create more targeted and diverse samples. Inspired by this, Cho et al. [44] proposed a stain style transfer (SST) model based on cGAN, using grayscale images as input and a classifier to improve performance. The SST model consists of two main components: the stain style generator and the discriminator. Unlike traditional generators that use noise vectors as input, the stain style generator takes images as input. Additionally, the classifier plays the role of the discriminator in this architecture, not only evaluating the output of the stain style generator but also acting as a feature extractor. It participates in extracting features to calculate feature-preserving loss, which optimizes the retention of tissue structure. By incorporating additional conditional information, the model can more finely control the image generation process, thus producing images that meet specific staining standards and requirements. However, since the test images need to be converted to grayscale before performing stain style transfer, this process may result in the loss of some critical information.

#### 5.1.2. Other Methods

**Frequency domain method:** Frequency domain processing techniques [53] offer advantages in digital image processing that differ from traditional spatial domain methods, particularly excelling in analyzing frequency distribution and enhancing image filtering. By separating an image’s low-frequency and high-frequency components, this technique allows for more precise adjustments to image features, thus improving the accuracy and efficiency of image processing. Li et al. [45] introduced frequency domain processing techniques into stain normalization and proposed the fourier stain normalization (F-SN) and fourier stain augmentation (F-SA) methods. These methods apply Fourier transform to convert images from the spatial domain to the frequency domain, and they combine deep learning models to optimize the image processing workflow.

**Random stain augmentation and normalization:** Traditional stain normalization methods often assume a linear relationship between color spaces, which can result in unrealistic color transformations. To address this issue, Wang et al. [46] developed RandStainNA++, a technique that combines stain normalization with stain augmentation. This method applies random stain normalization and augmentation in randomly selected color spaces, independently managing variations in the foreground and background of the image. Additionally, they introduced self-distillation techniques to improve the model’s ability to learn stain transformation invariance, thus enhancing its generalization capability. However, this dual forward propagation process may lead to an increase in training time.

**Vision Transformer (ViT):** In recent years, Transformers [54] have achieved significant success in the field of Natural Language Processing (NLP). Dosovitskiy et al. [55] introduced them to computer vision, proposing the ViT. The Swin Transformer (Shifted Window Transformer) [56] improved upon ViT by incorporating a sliding window mechanism, hierarchical design, and relative position encoding, which significantly enhances computational efficiency and model performance while retaining the global information capture capability of the Transformer. Kablan et al. [47] innovatively applied ViT to the field of stain normalization, proposing the StainSWIN model. This model combines the advantages of Swin Transformer and super-resolution architectures [57], improving the performance of stain normalization tasks. However, the StainSWIN model is currently limited to performing one-to-one stain mapping, and it still requires retraining when encountering new input domains.

#### 5.1.3. Data Partitioning Strategies

In supervised stain normalization methods, proper partitioning of training, validation, and test sets is crucial for ensuring model generalizability and preventing overfitting. Typically, 60–70% of the data are allocated to the training set to comprehensively learn diverse staining variations and tissue structural features. A validation set comprising 10–20% of the data is maintained to monitor the training process and optimize hyperparameters, while the remaining 10–20% serves as an independent test set for objective performance evaluation.

Different strategies should be adopted for datasets of varying scales: for small-scale datasets (e.g., MITOS-ATYPIA-14), k-fold cross-validation (k = 5 or 10) is recommended to enhance data utilization efficiency, whereas for large-scale datasets (e.g., CAMELYON 17), fixed-ratio partitioning generally suffices. It is particularly important to emphasize that the test set must include representative samples with staining variations and preferably originate from independent data acquisition environments to effectively validate the model’s robustness in real-world applications. This systematic data partitioning approach not only applies to conventional supervised learning but also establishes a foundation for evaluating subsequent unsupervised and self-supervised methods.

In the field of stain normalization, supervised learning methods have shown significant advantages in terms of accuracy and adaptability to specific targets, especially when training models with large amounts of labeled data. These methods enable models to better adapt to specific staining styles or features and allow for direct validation of model performance using known labels. However, this approach also faces notable drawbacks, such as its heavy reliance on large amounts of labeled data, which is difficult and costly to obtain in the medical field. Additionally, there is a risk of overfitting and insufficient generalization, as well as the potential impact of labeling errors. Furthermore, supervised learning for high-dimensional medical images requires substantial computational resources and time.

### 5.2. Unsupervised Methods

Unsupervised learning is a method that trains models using unlabeled data, where the model learns from the inherent structure and features of the data without relying on manually annotated labels [58]. In the field of stain normalization, labeled data are often difficult to obtain, which has fueled the development of unsupervised learning methods. Researchers frequently use unpaired images from two domains for training, often utilizing generative models such as autoencoders or GANs. These models can learn the underlying distribution of the data, thereby generating histopathological images with unified staining characteristics. Table 3 summarizes unsupervised stain normalization methods.

#### 5.2.1. Generative Adversarial Network (GAN) Methods

Zanjani et al. [59] developed a parametric, fully unsupervised generative model for stain normalization. Since deep convolutional neural networks (DCNNs) can approximate image data distribution in nonlinear color spaces, they replaced the latent variables of the source image with latent variables extracted from template images, enabling the model to generate copies of the source image with new colors while preserving essential tissue structures. However, this method primarily relies on specific colors from the target image rather than average colors, which can result in visually undesirable artifacts in standardized image processing [51].

Nazki et al. [60] adopted the method proposed by Choi et al. [79], which uses a single generator to learn mappings between different domains. Given that the original method struggled to preserve the content of WSIs when transforming input styles, they introduced an auxiliary feature extraction network to better retain fine anatomical structures. Additionally, they introduced perceptual loss to reduce the perceptual differences between real and generated images. While their method could normalize unseen domains, the multi-domain stain transfer process resulted in relatively slow training speeds.

**cGAN:** Federated Learning (FL) [80] methods use the parameters of local models to train a global model while ensuring data privacy and security [61]. In this context, Shen et al. [61] proposed a novel cGAN with a coordinated generator and multiple distributed discriminators to address stain style standardization in multi-client environments. This framework was implemented within the FL paradigm, effectively preserving data privacy and security. Furthermore, the introduction of a novel time self-distillation regularization strategy enhanced the consistency and stability of training in distributed systems.

**Figure 5:**
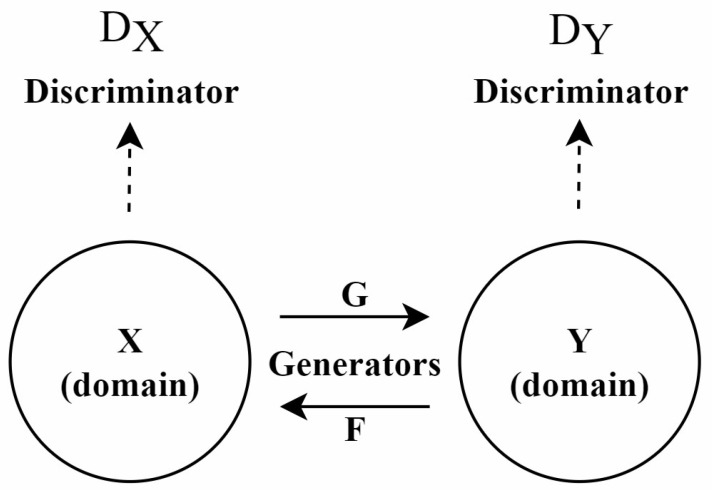
Architecture of the CycleGAN model, adapted from [81].

**CycleGAN:** Since paired training data are difficult to obtain in many practical scenarios, this has significantly limited the application of Pix2pix. To address this issue, CycleGAN (Cycle-Consistent GAN) [81] was proposed, particularly suited for environments lacking paired data. The architecture of this model is shown in Figure 5.

**Figure 6:**
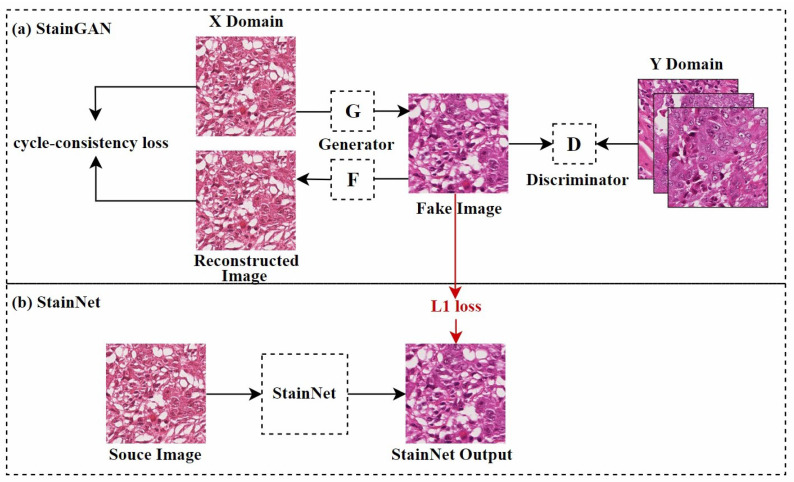
StainGAN and StainNet frameworks, adapted from [62,65]. StainGAN requires bidirectional mapping; the figure shows one direction of the mapping.

Shaban et al. [62] first proposed a CycleGAN-based model named StainGAN, as shown in Figure 6a. This model uses cycle consistency loss, which requires that when an image is transformed from one domain to another and then back to the original domain, the resulting image should remain similar to the original, thus preserving the tissue structure of the source image. Later, Kang et al. [65] used StainGAN as a “teacher model”, combining distillation learning [82] and a 1×1 CNN to reduce the computational cost of normalization, as shown in Figure 6b. This method learned the color mapping rules by analyzing the entire dataset and adjusted color values pixel by pixel, effectively controlling the network’s size and reducing visual artifacts during style transfer. Similarly, Lee et al. [66] used an improved U-Net [83] architecture, combining teacher–student learning strategies. They trained U-Net to perform stain normalization using paired datasets generated by the trained CycleGAN. Compared to traditional GAN techniques, this method used a simpler neural network structure, resulting in lower computational costs during training and inference.

Zhou et al. [12] proposed an enhanced CycleGAN method called color normalization GAN (CNGAN). This method calculates the stain color matrix for each H&E image in the training set, providing the generator with novel auxiliary inputs. Cai et al. [63] introduced transitive adversarial networks (TAN), an unsupervised CycleGAN-based framework for stain style transfer. They improved CycleGAN’s U-Net generator by increasing the number of downsampling and upsampling layers from 4 pairs to 8 pairs, enabling the network to capture higher-level semantic information and produce more detailed contextual features.

In the field of computer vision, the self-attention mechanism [84] enables a deeper understanding of complex visual content, allowing the model to simultaneously focus on all areas of the image and capture long-range dependencies and relationships between different regions. Thus, Shrivastava et al. [64] proposed a self-attentive adversarial stain normalization (SAASN) method, incorporating a self-attention mechanism [84] into CycleGAN to retain local context. This mechanism allows the network to effectively recognize the spatial relationships between different regions of the image, synthesizing images with finer details. Moreover, this improvement helps preserve the structural integrity of biological tissue features during style transfer and supports stain normalization from multiple source domains to a single target domain.

In 2015, He et al. [85] proposed residual learning, primarily used to build deeper neural networks while alleviating the degradation problem in deep network training. De Bel et al. [9] improved the original CycleGAN by introducing residual learning [85]. They modified the generator network by adding skip connections from the original image to the final output. This modification shifted the generator’s task from rebuilding the image from scratch to learning the residuals between different domains, allowing the generator to focus only on domain adaptation while ensuring the integrity of the image morphology.

Following StainNet [65], Kang et al. [16] further proposed a parameter-variable stain normalization network (ParamNet) and its corresponding adversarial training framework. ParamNet consists of a parameter prediction sub-network and a color mapping sub-network, with the parameters of the color mapping sub-network automatically adjusted by the parameter prediction sub-network for each input image. Notably, the color mapping sub-network employs a full 1×1 convolutional structure, similar to StainNet [65], which significantly improves computational efficiency. The proposed adversarial training framework introduces a texture module to resolve the mismatch between weak generators and strong discriminators.

Baykal et al. [67] proposed a CycleGAN-based regional realness-aware generative adversarial network (RRAGAN) for stain normalization. In their PatchGAN generator, a regional realness-aware mask generated by the convolution layer output guided the generator to focus on incorrect regions of the input image. This method effectively enhanced the realism of the images by concentrating on the specific areas of the image that appeared less realistic.

**Figure 7:**
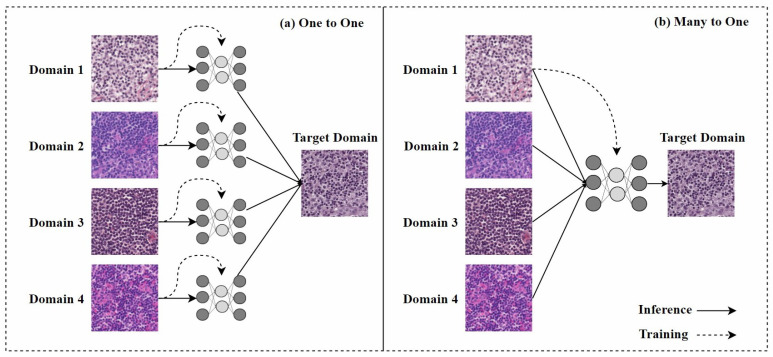
(**a**) One-to-one stain transfer method, where a separate model must be trained for each stain; (**b**) many-to-one stain transfer method, where only one model needs to be trained to normalize any H&E stain of the same tissue. The figure is adapted from [68].

In the past, one-to-one stain models required retraining when encountering staining from a new domain, as shown in Figure 7a, which limited their practical application. Therefore, research on many-to-one stain models has become a current focus. Once trained, many-to-one models can normalize multiple staining styles to the same target domain, as shown in Figure 7b.

Hetz et al. [68] proposed a many-to-one stain normalization method based on CycleGAN, called MultiStain-CycleGAN. They modified CycleGAN to standardize images from different sources without retraining the model. By converting RGB images into grayscale, they simplified the stain normalization problem, allowing the developed network to reconstruct images from grayscale images with varying contrasts. However, the conversion from RGB to grayscale during this process resulted in the loss of some important information.

While CycleGAN has made significant contributions to the stain normalization field, it also has some limitations. As a GAN, CycleGAN may introduce unnecessary artifacts or irrelevant tissue structures during the generation process, which could mislead doctors or downstream algorithms. Additionally, although CycleGAN does not rely on paired training data for domain conversion, its generalization ability remains limited. This is especially true when handling new images that differ significantly from the training set distribution, where its performance may fall short.

#### 5.2.2. Other Methods

**Autoencoder:** An autoencoder [86] is an unsupervised learning model typically used for dimensionality reduction and feature extraction. It consists of two parts: an encoder that compresses the input data into a low-dimensional representation, and a decoder that reconstructs the original data from the low-dimensional representation. By minimizing reconstruction error, an Autoencoder can learn latent feature representations of the data.

The Sparse AutoEncoder (SAE) [87] is an improved version of the Autoencoder that introduces a sparsity constraint during encoding. This sparsity constraint causes most hidden units to have activation values close to zero, activating only a small number of hidden units that are most relevant to the input data. This mechanism captures important features in the data, improving the model’s generalization ability and feature extraction performance. Inspired by SAE, Janowczyk et al. [69] proposed a stain normalization method called StaNoSa based on SAE. This method automatically divides the image into different tissue types and establishes tissue-specific correspondences between the target and template images, overcoming the limitations of traditional methods. This approach works because similar tissue types cluster together in the learned feature space. Compared to global approaches that consider all pixels, StaNoSa handles each tissue section separately, allowing for more refined adjustments to the color space. However, since multiple image processing steps are involved, some important information may be lost.

Jia et al. [70] extended the advantages of adversarial transfer learning and autoencoder structures by proposing the adversarial stain normalization network (ASNN). This network achieves stain normalization in histopathological images by aligning the distribution differences in latent features. In this process, the stain decoder learns the transformation rules from texture to stain by minimizing the reconstruction error of the target image, ensuring that the generated image has a consistent staining style while retaining the original texture information.

**Gaussian mixture model:** The gaussian mixture model (GMM) [88] is a statistical model used to represent the probability density function of multiple Gaussian distributions. It is a mixture model formed by linearly combining several Gaussian distributions with certain weights. Zanjani et al. [71] proposed an unsupervised probabilistic method that integrates convolutional neural networks (CNNs) and GMMs into a unified framework. Compared to traditional GMM-based methods, this approach clusters data based on the morphology and appearance of tissue structures and applies multivariate GMM fitting. In cases of strong stain variation, this method demonstrates greater robustness than standard GMMs, as the GMM fitting process accounts for the appearance of tissue structures in the image density channels.

**Figure 8:**
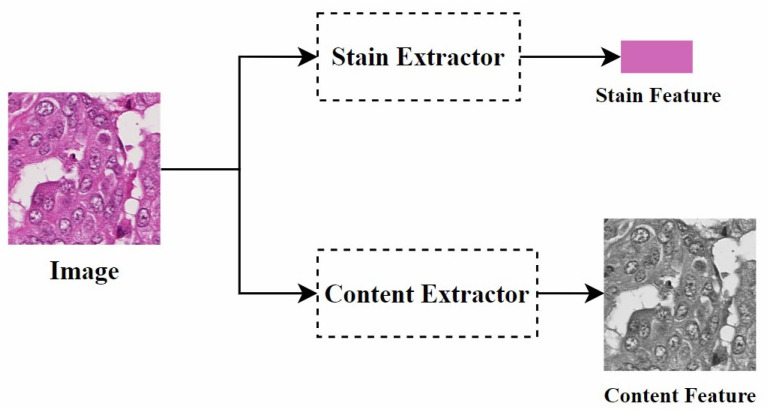
Disentangled representation of the concept diagram.

**Disentangled representation:** Disentangled representation in images refers to using specific models and algorithms to separate different features or attributes within an image so that these features can be independently manipulated and analyzed. Specifically, as shown in Figure 8, by separating the staining and content structure of a histopathological image, when processing the image, we can adjust the color style independently without changing the shape of the objects. This allows the image to present different staining effects while retaining its original structure.

Inspired by multimodal unsupervised image-to-image translation (MUNIT) [89], Xiang et al. [72] proposed a multi-domain stain normalization model. The main idea is to disentangle the representations of content and style. The method assumes that the latent space of histopathological images can be divided into a content space and a style space, where the content space contains structural information and the style space involves staining color appearance. The key challenge is learning to disentangle and represent the content and style spaces, ensuring that the content space remains consistent across different domains while the style space varies by domain. To achieve this, the style of an image from one domain can be combined with the content of an image from another domain, generating stain-normalized images through adversarial training.

Similarly, using disentangled representation [90], Moghadam et al. [73] were inspired by [91] to disentangle color variation features and structural features. The color variation features were used as cGAN encodings for stain conversion. They built two cGAN-based models to explore the role of feature disentanglement in the stain conversion process. These models covered both stain transfer and autoencoder functionality. The first model, called the one-to-one transfer (OOT) model, is an extension of the model in [91]. The generator of this model is divided into a shared branch and a dedicated branch to separate style and structural features, with the structural branches of different generators sharing weights. The second model, called the many-to-many transfer (MMT) model, is an improvement on the OOT model. Unlike OOT, the MMT model uses a single generator to handle all styles, similar to the StarGAN architecture [79]. However, the MMT model also includes an autoencoder component to maintain structural integrity and does not rely on source-style labels. Therefore, the model supports many-to-one stain transfer, requiring only the target-style label. However, the use of multiple loss functions to achieve many-to-many stain conversion leads to longer training times.

Mahapatra et al. [74] also used disentangled representation [90] and proposed a new hybrid latent model called TredMiL (Truncated normal Mixture-based Latent model), which disentangles color information and stain density information in histopathological images. They used truncated normal distributions as the latent priors for color appearance and stain density. The proposed hypothesis is that the latent color information extracted by the color appearance encoder is independent of the stain boundary information extracted by the stain density encoder. To capture disentangled color appearance and stain boundary information, the designed model incorporates generation and reconstruction modules that jointly form the model’s objective function.

**Capsule networks:** Capsule networks [92], proposed by Geoffrey Hinton and his team in 2017, improve upon traditional CNNs by using vector outputs and dynamic routing mechanisms, particularly in handling spatial relationships and pose variations. Inspired by capsule networks, Zheng et al. [75] proposed a novel stain normalization module based on capsule networks and the corresponding dynamic routing algorithm, called the stain standardization capsule (SSC). The main idea behind this method is to provide a unified stain separation result for images with different staining styles, thereby achieving stain normalization. Unlike existing methods [35] that typically address specific stain separation problems for each image or WSI, this method learns a set of stain separation parameters from the overall color distribution of the training data. During the application phase, through dynamic routing (DR) operations, the model can dynamically select the most suitable stain separation parameters for each image, improving the model’s robustness.

**Invertible neural networks:** When convolutional neural networks process images, some critical depth information may be lost. To address this issue, invertible neural networks (INNs) [93] can learn a reversible representation to reduce information loss under specific conditions. Based on this, Lan et al. [76] proposed an unsupervised color normalization method based on channel attention [94] and long-range residuals [85], using INN technology to transfer stain styles. This method maintains semantic consistency of tissue across different hospitals or centers.

**Diffusion Model:** Recently, diffusion probabilistic models (DPMs) [95], as a powerful generative model, have gained widespread attention. The basic principle is to simulate the physical diffusion process, treating data generation as a reverse diffusion process [96]. The denoising diffusion probabilistic model (DDPM) [20] is a variant of DPM that restores and generates data through a process of gradually adding and removing noise. Inspired by this, Shen et al. [77] proposed StainDiff, the first pure DDPM [20] designed for stain transfer. Unlike existing Diffusion Models, StainDiff can learn from unpaired histological images. Compared to GAN-based methods, StainDiff’s training of the additional discriminator comes at no extra cost and avoids the difficulties associated with posterior probability alignment in AE-based methods.They also proposed a self-ensemble scheme to further improve and stabilize StainDiff’s performance in style transfer.

**Figure 9:**
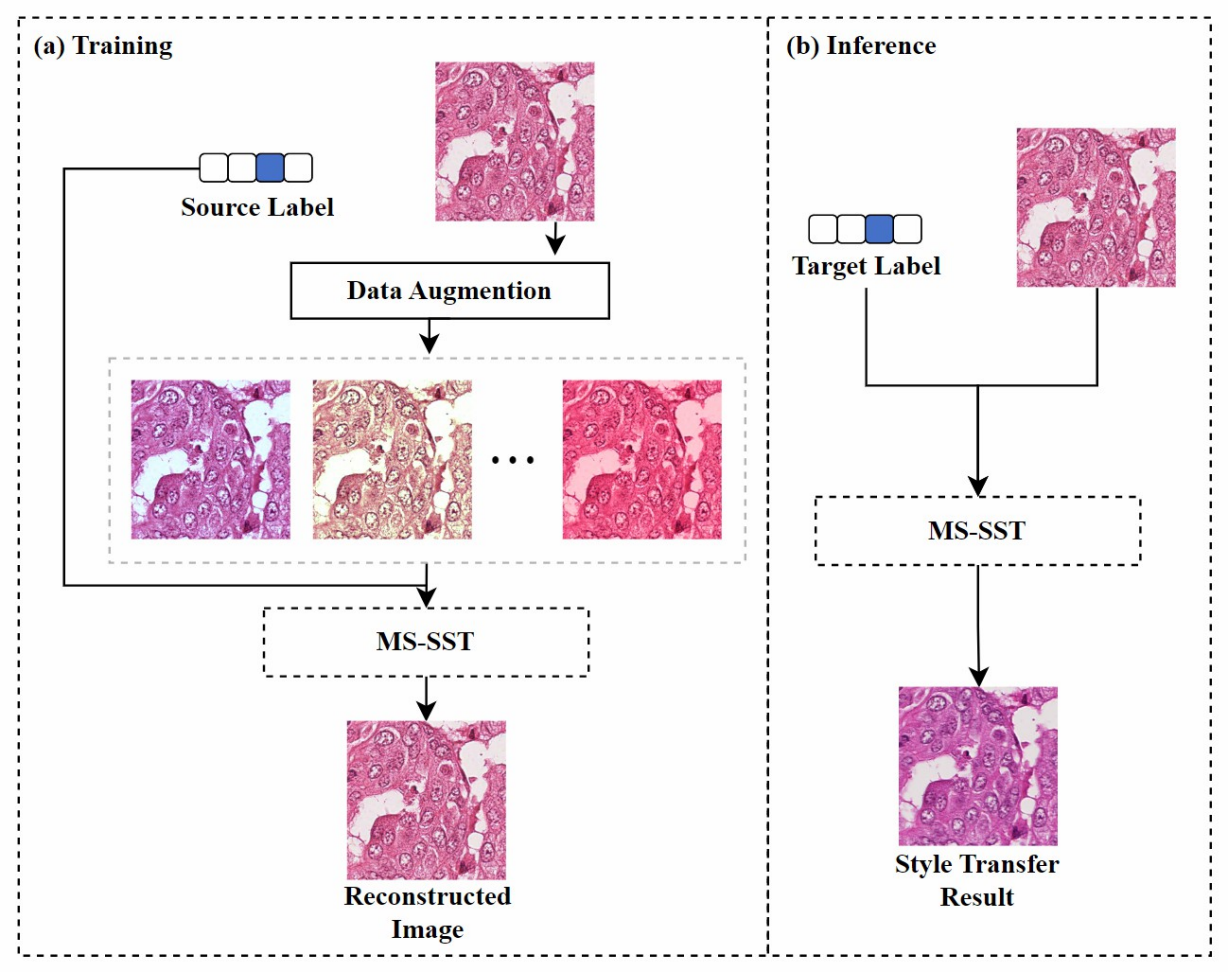
The MS-SST model’s training and inference framework. (**a**) shows the process of reconstructing a disturbed source image using MS-SST. (**b**) shows the process of transferring the source image to the target label domain using MS-SST. Figure adapted from [78].

**Image reconstruction-based:** Inspired by the SinIR training protocol [97], Kweon et al. [78] proposed a model based on multi-domain single image reconstruction stain style transfer (MS-SST). The model is trained using a reconstruction-based learning framework that can translate between multiple domains using a single training image from each domain. As shown in Figure 9, during the training phase, the model’s goal is to restore the damaged source image to its original form. The geometrically and color-enhanced original image, along with the source label, are fed into the model, which uses perceptual loss [98] and structural similarity index loss to retain the semantic information of the source image. During inference, the model maps the input source image to the specified target domain using the domain label vector.

Unsupervised methods eliminate the reliance on paired data and do not require classification or segmentation labels. This is especially important in the field of histopathological imaging, where expert annotations are costly and time-consuming. However, unsupervised methods in stain normalization still require at least two domains of data for training, which often makes it impossible to transfer stains to unseen domains. Additionally, the unsupervised normalization process may unintentionally alter or lose important tissue structure information, potentially affecting subsequent image analysis and diagnostic accuracy.

### 5.3. Self-Supervised Methods

Self-supervised learning is a method that uses the structure or attributes of the data themselves to generate labels and train models with these generated labels [99]. In the field of stain normalization, the introduction of self-supervised learning addresses the challenge of data labeling. This approach enables models to automatically extract useful information from large amounts of unlabeled data, reducing the reliance on expert annotations. Table 4 summarizes self-supervised stain normalization methods.

#### 5.3.1. Generative Adversarial Network (GAN) Methods

Zhao et al. [100] developed a self-supervised learning model named RestainNet, designed as a digital re-staining tool. The model learns how to stain grayscale images with two digital stains, hematoxylin and eosin. To ensure the accuracy of the staining process, they introduced a new stain loss function, which compares the digital H&E stains extracted from the RestainNet-generated re-stained images and the original images. Since they used a self-supervised learning method, there was no need to collect paired images for network training, which improved the feasibility of the model in practical applications.

**Figure 10:**
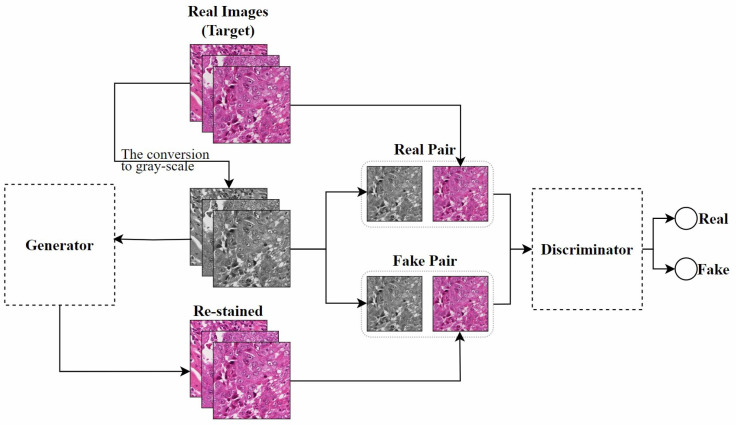
The Pix2pix-based STST method proposed by Salehi et al., adapted from [14].

**Pix2pix:** Isola et al. [107] studied the application of cGAN in image-to-image translation problems and proposed the Pix2pix model. This model requires paired images for training, meaning that each input image has a corresponding expected output image, which is a key prerequisite for training this model. Inspired by their work, Salehi et al. [14] proposed a stain-to-stain translation (STST) method based on Pix2pix. Since it is often difficult to obtain paired original and corresponding transformed images in practical applications, as shown in Figure 10, they trained on paired patches of histopathological images and their corresponding grayscale images to achieve a many-to-one stain transfer method. However, converting to grayscale may result in the irreversible loss of staining information in the input images, leading to reduced image contrast. This could negatively affect the accuracy of pathological detection and classification.

Inspired by the STST method, Cong et al. [101] proposed a texture-enhanced Pix2pix generative adversarial network (TESGAN) for generating high-quality stain-normalized images. The key improvement lies in using the hematoxylin component of H&E-stained images as the input to the generator, as the hematoxylin component provides higher contrast between the nucleus and cytoplasm, making it more effective than traditional grayscale images. Additionally, they introduced a combination of content loss and L1 loss to enhance the accuracy of the generated image’s color patterns while preserving the complex features of the input images, including both high-level and low-level features.

**CycleGAN:** Mahapatra et al. [102] proposed a self-supervised method that integrates semantic guidance into a CycleGAN-based stain normalization framework. Specifically, their framework incorporates semantic information from different layers of a pre-trained semantic network and a stain normalization network to preserve detailed structural information. Additionally, their method does not require manually segmented maps, reducing dependence on labeled datasets.

#### 5.3.2. Other Methods

**Coupling network:** In earlier stain normalization research [8], stain normalization was achieved by training a network to map stain-augmented images back to their corresponding original images. The auxiliary task in this process was to learn how to convert the stain-augmented images back to the original images, while converting the staining style of test images to match that of the training data was considered the downstream task. Gehlot et al. [103] used this approach and proposed a novel coupling network that combines two U-Net-like architectures and employs a self-supervised learning method for training. The first sub-network (N1) learns the identity transformation, while the second sub-network (N2) learns to perform stain normalization. By coupling N1 and N2, N2 can better learn the stain normalization task while identifying the features necessary for reconstructing the image. Similarly, N1, through its coupling with N2, learns to extract relevant features for reconstruction, ensuring consistency in stain color changes. This coupling of the two sub-networks improved the performance of subsequent classification tasks.

**Contrastive learning:** Although the following two methods fall within the GAN category, they are described here due to their core technology being contrastive learning.

In recent years, some researchers have introduced contrastive learning into the stain normalization field. Contrastive learning is a self-supervised learning method that trains by treating different transformations of the same image as positive sample pairs to learn image feature representations [108]. In this context, Ke et al. [104] introduced color variation constraints while training a color transfer GAN and combined a contrastive learning-based loss function to effectively retain key invariant information in phenotype recognition. They also grouped patches with consistent phenotypes across different tumors to analyze the shared characteristics of these patches.

Similarly, Gutiérrez et al. [105] proposed a contrastive learning-based stain normalization method, which is an improvement of Park et al.’s [109] unpaired image-to-image method. They used adversarial loss to force the generator’s output to resemble images from the target domain while maintaining content consistency through the contrastive learning objective function. Although this method used contrastive learning to achieve transformations between different staining styles, if the style of the image to be transformed differs greatly from the styles in the training set, the conversion quality may be affected.

**Figure 11:**
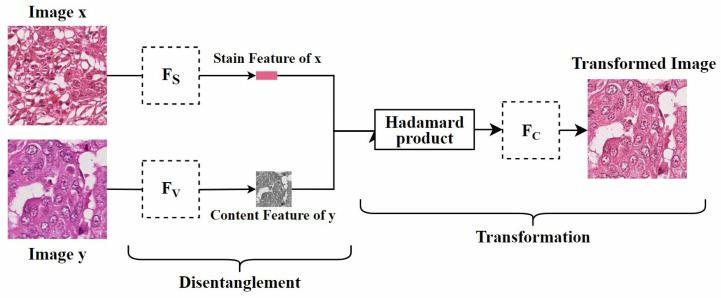
Self-supervised Disentanglement Network. $F_{S}$ is used to extract the stain features of the image, $F_{V}$ is used to extract the content features of the image, and $F_{C}$ is used to reconstruct the image. The figure is adapted from [5].

**Disentangled representation:** Since traditional GAN models rely on explicit source and target domains, they cannot handle unknown domains. To address this, Ling et al. [5] proposed a self-supervised disentanglement network (SDN) for domain-independent optimization and arbitrary-domain stain transfer. They also introduced a self-supervised learning strategy, based on stain and content consistency, to augment training domains through image enhancements (including spatial and color augmentations). The goal was to maximize the similarity between the enhanced images and their synthesized results. As shown in Figure 11, SDN extracts content and stain features by decomposing the enhanced images and transfers the stain style to the target domain by exchanging these stain features. Since SDN’s training process is independent of specific domain data, it can handle previously unseen domains, which is significant for practical applications.

**Diffusion Model:** Similarly, to address the problem of GAN methods being unable to directly apply to unknown domains, Jewsbury et al. [106] proposed StainFuser. This model is based on the Conditional Latent Diffusion (CLD) [110] architecture and treats stain normalization as a style transfer task. To train StainFuser, the authors employed neural style transfer [111] to generate transformed versions of each pair of source and target images. Previous research [86] has shown that, compared to GANs, Diffusion Models perform better in terms of quality and training stability.

Self-supervised learning is particularly important in the field of histopathological stain normalization, as it significantly reduces dependence on expert-labeled data and utilizes large amounts of unlabeled data to improve model generalization. However, the performance of self-supervised methods may not be as strong as supervised learning, especially when dealing with regions with significant tissue structure differences. Additionally, these methods heavily rely on the quality and diversity of the unlabeled data. Therefore, researchers need to design effective self-supervised learning tasks to improve the quality of stained images.

## 6. Discussion and Future Directions

### 6.1. Current Challenges

Although significant progress has been made in stain normalization through deep learning technologies, several challenges and unresolved issues remain.

#### 6.1.1. Difficulty in Data Acquisition

In the field of histopathology, training supervised learning models requires a large amount of labeled data. However, it is quite difficult to obtain these data. Among them, paired datasets used to evaluate the performance of stain normalization are particularly scarce. This is because obtaining paired datasets usually requires using multiple scanners to scan the same slide and align the pixels through image registration, which places high demands on the equipment and faces many challenges in practical operation.

The currently widely used MITOS-ATYPIA-14 dataset has certain limitations. It has a limited number of slides and only focuses on specific histological images of breast cancer. It is difficult to comprehensively cover various types of histopathological images with different types and staining styles. As a result, when using this dataset for stain normalization research, it is difficult to fully verify the generalization ability of relevant research results, and it is impossible to determine whether these research results are equally effective on other histopathological images.

In addition, the existing method of obtaining paired data using scanners can only reflect the staining differences caused by scanners and cannot reflect the impacts brought about by inconsistencies in parameters such as staining time, temperature, and reagent concentration during the tissue preparation process. The changes in these parameters can significantly affect the staining effect, but the current dataset acquisition method fails to take them into account.

In view of the above problems, researchers need to actively explore new directions for datasets used to evaluate stain normalization, and seek more effective and representative methods for obtaining datasets to promote the development of stain normalization research in the field of histopathology.

#### 6.1.2. Limitations of Evaluation Metrics

Existing evaluation metrics have certain limitations, and there is a need to develop more suitable metrics for this field to more accurately assess the performance of stain normalization algorithms. For instance, PSNR only considers pixel-level errors between two images at the same positions without accounting for human visual characteristics, which may result in discrepancies between its evaluation results and human visual perception. In some cases, even if the PSNR value is high, the image may still exhibit unnatural color or structural issues, which may not be accurately reflected by PSNR. SSIM primarily focuses on changes in structural information, but in some cases, it may not fully assess the quality of the normalized image. Additionally, methods that perform better on stain normalization evaluation metrics may not necessarily show outstanding performance in downstream tasks. Therefore, there is a need to explore a metric that can comprehensively evaluate both the stain normalization performance and the performance in downstream tasks.

#### 6.1.3. Artifacts and Information Loss

Within the current research scope of stain normalization, existing methods have numerous limitations. In certain specific situations, existing stain normalization methods find it difficult to completely eliminate the occurrence of artifacts. There are multiple mechanisms behind this. On the one hand, algorithms struggle to accurately simulate complex color distributions. For example, in CycleGAN [81], during the adversarial training between the generator and the discriminator, the generator may produce images with artifacts in an attempt to deceive the discriminator. On the other hand, the limited ability of neural networks to handle high-dimensional color spaces also contributes to the generation of artifacts.

Meanwhile, some methods process images by converting them into grayscale, an operation that leads to the loss of crucial information. Moreover, some methods may also exhibit the phenomenon of over-normalization, which greatly restricts the accuracy and reliability of deep learning methods in practical applications.

Artifacts have negative impacts of varying degrees on different downstream tasks. In the pathological diagnosis process, artifacts can cause doctors to misinterpret tissue structures, and further lead to incorrect judgments about the presence or stage of diseases. In image-based classification and segmentation tasks, artifacts interfere with the accuracy of feature extraction, reducing the performance of machine learning models and making it difficult to obtain reliable results.

For instance, when using CycleGAN for stain normalization, although it can achieve stain style conversion to a certain extent, the unnecessary artifacts introduced during the generation process will not only mislead doctors but also disrupt the operation of downstream algorithms. Similarly, the method of converting images into grayscale for processing will result in irreversible loss of color information, such as a decrease in image contrast, which has an adverse effect on the accuracy of pathological detection and classification.

To effectively address the issue of artifacts, future research can be carried out in the following directions: Firstly, optimize the network architecture. Taking GAN-based methods as an example, design more advanced generators and discriminators to enable them to better capture color features and reduce the probability of artifact generation. Secondly, explore new loss functions to achieve a better balance between color normalization and artifact suppression. Thirdly, integrate the prior knowledge of tissue staining into the model training process to help the model better understand and cope with actual staining variations, thereby minimizing artifacts to the greatest extent possible.

### 6.2. Future Directions

#### 6.2.1. Development of Lightweight Models

As deep learning-based stain normalization methods are increasingly applied in practical scenarios, developing lightweight models suitable for deployment will become an important trend. For example, utilizing model compression techniques, such as knowledge distillation [82] and automated model search [112], can reduce the number of parameters and computational load, allowing models to run efficiently on resource-constrained devices. Additionally, exploring the use of more efficient neural network architectures, such as EfficientNetV2 [113] and GhostNet [114], can help reduce model complexity and improve deployment efficiency.

#### 6.2.2. Arbitrary-Domain Stain Normalization

Currently, most stain normalization models are limited to processing only the data domains encountered during training. However, in clinical practice, stain variations are far more diverse, making it crucial to develop models capable of normalizing images from previously unseen source domains to a consistent target domain. For instance, the self-supervised disentanglement network proposed by Ling et al. [5] employs an image disentanglement technique to separate stain and structural features. Through training with spatial and color augmentation, the model achieves normalization across arbitrary source domains. Nevertheless, the current stain accuracy still requires improvement, underscoring the importance of further research in this area.

#### 6.2.3. Reinforcement Learning and Optimization Algorithms

Recent studies have demonstrated the potential of Reinforcement Learning (RL) for stepwise optimization in image-to-image translation (e.g., the lightweight RL-I2IT framework proposed by Wang et al. [115]). Since staining normalization can be formulated as an image-to-image conversion problem, reference can be made to their proposed framework. The technique can gradually optimize the conversion strategy by defining dyeing parameters (e.g., H&E concentration, optical density) as state spaces. The application of RL algorithms allows models to automatically learn optimal strategies during stain normalization, such as selecting the best parameter settings or processing steps. Meanwhile, advanced optimization algorithms like Adabelief and Ranger can significantly improve training efficiency and convergence speed. Despite its great potential, practical applications of RL in pathological images still face challenges including reward function design (e.g., quantification of nuclear morphology preservation) and multicenter generalization validation.

## 7. Conclusions

During the preparation of digital histopathological slides, variations in staining protocols and scanners often lead to changes in the staining appearance of pathological images, resulting in color distribution shifts. These shifts can negatively impact the performance and robustness of CAD systems. To address this issue, researchers have proposed stain normalization techniques to standardize the color appearance of images, thereby reducing the impact of color distribution shifts on model performance.

In this study, we systematically reviewed the latest advancements in deep learning-based stain normalization of histopathological images, including supervised, unsupervised and self-supervised stain normalization methods. We further categorized them based on core technologies, highlighting their main contributions and limitations. Currently, the application of deep learning in stain normalization shows great potential, especially in addressing stain variations and preserving tissue structure. However, this technology also faces several challenges and unresolved issues, such as difficulties in data acquisition, limitations of evaluation metrics, and problems related to artifacts and information loss.

To address the current challenges, we propose several future research directions for deep learning in stain normalization: First, the development of lightweight models by utilizing model compression techniques and efficient architectures to reduce parameters and computation load, improving deployment efficiency. Second, the development of models capable of normalizing images from previously unseen source domains to a consistent target domain and enhancing their accuracy, considering the limitations of current models in handling diverse clinical stain variations. Third, the application of reinforcement learning and advanced optimization algorithms to enable models to automatically learn optimal strategies, improving training efficiency and convergence speed.

Despite the challenges, deep learning-based stain normalization in histopathological images remains a promising research area. With continuous exploration and innovation, it is expected to significantly improve the diagnostic accuracy and efficiency of major diseases, including cancer, and have a profound impact in the future.

## Figures and Tables

**Table 1:** Overview of datasets used for stain normalization, classification, and segmentation tasks.

Type	Dataset	Body Parts	Format	Data Volume	Image Details
Stain normalization	MITOS-ATYPIA-14 [10]	Breast	TIFF	Training: 11 WSIs Testing: 5 WSIs	Two sets of images with different staining styles from two scanners.
	SCC [30]	Canine cutaneous	TIFF	220 WSIs	44 samples scanned using 5 different scanners, resulting in 220 WSIs.
Classification	CAMELYON 16 [27]	Lymph node	TIFF	Training: 270 WSIs Testing: 130 WSIs	WSIs from two medical centers, different staining styles at each center.
	CAMELYON 17 [11]	Lymph node	TIFF	Training: 500 WSIs Testing: 500 WSIs	pN-stage classification of breast cancer metastasis from five centers.
	TUPAC 2016 [31]	Breast	SVS	Training: 500 WSIs Testing: 321 WSIs	Images from 73 breast cancer patients across three pathology centers.
	BACH ICIAR 2018 [32]	Breast	TIFF	400 patches	100 images each of normal, benign, in situ carcinoma, and invasive carcinoma.
	BreakHis [33]	Breast	PNG	Benign: 2480 patches Malignant: 5429 patches	9109 images from 82 patients. Image size: 700×460 px.
Segmentation	MICCAI2015 GlaS [28]	Colorectum	BMP	Training: 85 patches Testing: 80 patches	165 patches (775×522 px @0.62 μm/px, 20×), validated for gland segmentation [28].
	MoNegSeg [29]	7 organs	TIFF	30 patches	Includes seven organs: breast, kidney, liver, prostate, bladder, colon, and stomach.

WSIs: Whole-Slide Images.

**Table 2:** Supervised stain normalization methods. The “Cls” column and the “Seg” column indicate whether downstream classification or segmentation tasks are used to indirectly evaluate the performance of stain normalization, respectively.

Author	Model Name	Year	Technology	Journal/Conference	Datasets	Cls	Seg
Bentaieb et al. [40]	-	2018	GAN	*IEEE Trans. Med. Imaging*	MITOS-ATYPIA-14MICCAI’2015 GlaSprivate dataset	√	-
Nishar et al. [41]	HRNet	2020	GAN	MICCAI 2020	MITOS-ATYPIA-14	√	-
Liang et al. [42]	SSIM-GAN & DSCSI-GAN	2020	GAN	MLMIR 2020	CAMELYON 16	-	√
Kausar et al. [43]	SA-GAN	2022	GAN	*Appl. Sci.*	MITOS-ATYPIA-14TUPAC 2016BACH ICIAR 2018MICCAI’2015 GlaS	√	-
Cho et al. [44]	SST	2017	cGAN	arxiv	CAMELYON 16	√	-
Li et al. [45]	F-SN, F-SA	2023	Frequency domain method	IEEE BIBM 2023	NCT-CRC-HE-100KNONORMMoNuSeg	√	-
Wang et al. [46]	RandStainNA++	2024	Random stain augmentation and normalization	*IEEE J. Biomed. Health Inform.*	CAMELYON 17NCT-CRCMoNuSegprivate dataset	√	√
Kablan et al. [47]	StainSWIN	2024	ViT	*Eng. Appl. Artif. Intell.*	MITOS-ATYPIA-14MICCAI’2015 GlaS	-	√

**Table 3:** Unsupervised stain normalization methods. The “Cls” column and the “Seg” column indicate whether downstream classification or segmentation tasks are used to indirectly evaluate the performance of stain normalization, respectively.

Author	Model Name	Year	Technology	Journal/Conference	Datasets	Cls	Seg
Zanjani et al. [59]	-	2018	GAN	IEEE ISBI 2018	private dataset	-	-
Nazki et al. [60]	MultiPathGAN	2023	GAN	ACM/SIGAPP SAC 2023	private dataset	-	-
Shen et al. [61]	-	2023	cGAN	*IEEE Trans. Med. Imaging*	TCGA-COADCRC-VALHE-7KNCT-CRC-HE-100K	√	-
Shaban et al. [62]	StainGAN	2019	CycleGAN	IEEE ISBI 2019	MITOS-ATYPIA-14CAMELYON 16	-	-
Shaban et al. [62]	StainGAN	2019	CycleGAN	IEEE ISBI 2019	MITOS-ATYPIA-14CAMELYON 16	-	-
Zhou et al. [12]	CNGAN	2019	CycleGAN	MICCAI 2019	CAMELYON 16CAMELYON 17TUPAC 2016MITOS-ATYPIA-14MICCAI’15 GlaS	√	-
Cai et al. [63]	TAN	2019	CycleGAN	MLMIR 2019	MITOS-ATYPIA-14	-	-
Shrivastava et al. [64]	SASAN	2021	CycleGAN	ICPR 2021	MITOS-ATYPIA-14private dataset	-	-
De Bel et al. [9]	-	2021	CycleGAN	*Med. Image Anal.*	private dataset	√	-
Kang et al. [65]	StainNet	2021	CycleGAN	*Front. Med.*	MITOS-ATYPIA-14CAMELYON 16private dataset	√	-
Lee et al. [66]	-	2022	CycleGAN	IEEE BIBE 2022	private dataset	-	-
Kang et al. [16]	ParamNet	2023	CycleGAN	arxiv	MITOS-ATYPIA-14CAMELYON 16CAMELYON 17private dataset	√	-
Baykal et al. [67]	RRAGAN	2023	CycleGAN	*Neural Comput. Appl.*	MITOS-ATYPIA-14CAMELYON 16MICCAI2015 GlaS	√	-
Hetz et al. [68]	MultiStain-CycleGAN	2024	CycleGAN	*Med. Image Anal.*	CAMELYON 17 SCC	√	-
Janowczyk et al. [69]	StaNoSA	2017	AE	*Comput. Med. Imaging Graph.*	private dataset	-	-
Jia et al. [70]	ASNN	2022	AE	IEEE BIBM 2022	MITOS-ATYPIA-14CAMELYON 16	√	-
Zanjani et al. [71]	-	2018	GMM	MICCAI 2018	private dataset	-	-
Xiang et al. [72]	RRAGAN	2020	Disentangled representation	IEEE ICIP 2020	CAMELYON 17	-	-
Moghadam et al. [73]	OOT, MMT	2022	Disentangled representation	*Comput. Biol. Med.*	MITOS-ATYPIA-14CAMELYON 16DigestPath	-	-
Mahapatra et al. [74]	TredMIL	2023	Disentangled representation	*IEEE Trans. Med. Imaging*	UCSB DataCMU Data	√	-
Zheng et al. [75]	SSC	2021	Capsule network	*IEEE J. Biomed. Health Inform.*	CAMELYON 16ACDC-LungHPprivate dataset	-	-
Lan et al. [76]	-	2021	INN	IEEE Access	MITOS-ATYPIA-14	-	-
Shen et al. [77]	StainDiff	2023	Diffusion Model	MICCAI 2023	MITOS-ATYPIA-14TCGA	√	-
Kweon et al. [78]	MS-SST	2024	Image reconstruction-based	IEEE Access	MITOS-ATYPIA-14CAMELYON 17	-	√

**Table 4:** Self-supervised stain normalization methods. The “Cls” column and the “Seg” column indicate whether downstream classification or segmentation tasks are used to indirectly evaluate the performance of stain normalization, respectively.

Author	Model Name	Year	Technology	Journal/Conference	Datasets	Cls	Seg
Zhao et al. [100]	RestainNet	2022	GAN	*Comput. Electr. Eng.*	MITOS-ATYPIA-14TSR-CRCMICCAI’16 GlaS	√	√
Salehi et al. [14]	STST	2020	Pix2pix	MVIP 2020	MITOS-ATYPIA-14	-	√
Cong et al. [101]	TESGAN	2021	Pix2pix	IEEE ISBI 2021	TCGA LGGTCGA GBM	√	-
Mahapatra et al. [102]	SegCN-Net	2021	CycleGAN	MICCAI 2020	CAMELYON 16CAMELYON 17MICCAI’16 GlaS	√	√
Gehlot et al. [103]	AION	2021	Coupling Network	MLMI 2021	CAMELYON 17PCamData Science Bowl (DSB)CVC-ClinicDB (CVC)	√	√
Ke et al. [104]	-	2021	Contrastive Learning	MICCAI 2021	TCGA-COADTCGA-READTCGA-STADCAMELYON 16NCT-CRC-HE-100K-NORM	-	-
Gutiérrez et al. [105]	StainCUT	2022	Contrastive Learning	*J. Imaging*	MITOS-ATYPIA-14CAMELYON 16	-	√
Ling et al. [5]	SDN	2023	Disentangled Representation	*IEEE Trans. Med. Imaging*	MITOS-ATYPIA-14CAMELYON 17	√	-
Jewsbury et al. [106]	StainFuser	2024	Diffusion Model	arxiv	TCGA-STADTCGA-COADTCGA-READCoNIC	-	√

## Data Availability

The authors do not have permission to share data.

## Abbreviations

The following abbreviations are used in this manuscript:

**Abbreviation**	**Full Form**
H&E	Hematoxylin and eosin
CAD	Computer-aided diagnosis
OD	Optical density
GAN	Generative Adversarial Network
AE	Autoencoder
DM	Diffusion Model
WHO	World Health Organization
WSIs	Whole-Slide Images
PSNR	Peak Signal-to-Noise Ratio
MSE	Mean squared error
SSIM	Structural Similarity Index
NMI	Normalized Median Intensity
PCC	Pearson Correlation Coefficient
FSIM	Feature Similarity Index
GPU	Graphics Processing Unit
DCNN	Deep convolutional neural network
CNGAN	Color normalization GAN
TAN	Transitive adversarial network
SAASN	Self-attentive adversarial stain normalization
RRAGAN	Regional realness-aware generative adversarial network
SAE	Sparse AutoEncoder
StaNoSa	Stain normalization using sparse autoencoders
ASNN	Adversarial stain normalization network
GMM	Gaussian mixture model
OOT	One-to-one transfer
MMT	Many-to-many transfer
TredMiL	Truncated normal Mixture-based Latent model
SSC	Stain standardization capsule
INN	Invertible neural network
DPM	Diffusion probabilistic model
DDPM	Denoising diffusion probabilistic model
MS-SST	Multi-domain single image reconstruction stain style transfer
STST	Stain-to-stain translation
TESGAN	Texture-enhanced Pix2pix generative adversarial network
SDN	Self-supervised disentanglement network
CLD	Conditional Latent Diffusion
FL	Federated Learning
ViT	Vision Transformer
Swin Transformer	Shifted Window Transformer
HRNet	High-Resolution Network
DSCSI	Directional statistics-based color similarity index
SA-GAN	Stain acclimation generative adversarial network
cGAN	conditional Generative Adversarial Network
SST	Stain style transfer
F-SN	Fourier stain normalization
F-SA	Fourier stain augmentation
NLP	Natural Language Processing
CNN	Convolutional neural network
MUNIT	Multimodal unsupervised image-to-image translation
DR	Dynamic routing
N1	Sub-network 1 (identity transformation)
N2	Sub-network 2 (stain normalization)
